# Financial investment trust mechanism based on smart contract

**DOI:** 10.1371/journal.pone.0287706

**Published:** 2023-07-28

**Authors:** Wei Xiong, Danping Wan

**Affiliations:** Nanchang Institute of Technology, School of Business Administration, Nanchang, 330099, China; National University of Sciences and Technology NUST, PAKISTAN

## Abstract

In this paper, a financial investment trust solution based on smart contract is proposed to solve the distrust problem in financial investment caused by information asymmetry. By utilizing the functional attributes of blockchain-based smart contracts, the financial investment trust mechanism is established. By operating this mechanism, the financial investment information is received, stored, and processed, and the information is sent to the nodes in the blockchain. By devising the algorithms of "requesting financial investment service", "successful financial investment", "product contract fraud arbitration" and "investment contract fraud arbitration", the financial investment trust mechanism is achieved. By presenting the algorithms and their invoking processes, smart contracts for the solution are written and debugged. Finally, the smart contracts are tested and validated. The smart contract source code is available in GitHub.

## Introduction

Trust mechanism is usually defined as the relationship management mechanism among the parts that constitute and affect the mutual trust relationship in the enterprise system [1]. For users in the network environment, there are three trust mechanisms for online trading [2]: (1) Deterrence-type trust mechanism. Deterrence trust means that participants will complete their tasks according to their respective commitments because they are afraid of being punished. This trust model will make the cost of opportunism higher than its benefits and will play a role in the whole cooperation process, such as legal constraints. (2) Technology-type trust mechanism. Generally speaking, in the early stage of cooperation, due to the lack of understanding of each other’s credit information, mutual suspicion or opportunistic behavior is inevitable. Both parties are rational and will carefully consider the benefits and costs of trust. Therefore, trust is a market-oriented economic calculation, and its value depends on the difference between the benefits derived from trust and the cost of maintaining trust. It is the benefit that prevents the occurrence of dishonest behavior. (3) Understanding-type trust mechanism. In the process of cooperation, the trading parties have enhanced their understanding through formal and informal information communication. Both parties can understand and predict the behavior of the other party. As time goes by, both parties will continuously enhance their sense of trust based on the actual good performance of the other party. For example, users gradually form a sense of trust in the website through the use of the website and trading behavior.

In our daily life, there are many customers with financial investment needs. They make financial investments online through investment advisors of centralized financial institutions. However, the investment advisor’s advice is primarily for sales or marketing. The advice of investment advisors is passive, and those who are willing to believe can follow the advice and invest. There is no direct reward and punishment mechanism for investment advisor advice. The wages earned by investment advisors are essentially rebates or commissions given by the product side. Therefore, from the perspective of interests, the starting point of investment advisors is to sell more products, but most of the time they do not really stand on the position of customers. There will be a phenomenon that investment advisors encourage customers to conduct more frequent transactions, such as redemption/ purchase. This can easily lead to centralized financial institutions losing the trust of their customers. Furthermore, a centralized system has obvious disadvantages [3]: (1) If the system is attacked by hackers or other organizations, the destruction of the database will lead to the collapse of the entire system, and there is a possibility that all user information will be leaked or all money will be stolen. (2) There is information asymmetry between the centralized system and users, which may have potential adverse effects on user privacy and interests. In the centralized system, the user’s name, address, age, hobbies, economic status, living habits, social circle, and other private information will be acquired by the centralized system for data storage, analysis, and processing. This can easily cause user information to be leaked or used by people with ulterior motives. (3) The rights of the centralized system and users are asymmetric. If the centralized system is used to do bad things, the consequences can be severe. For example, the user’s data can be modified at will through the background. It is because of these issues that the centralized trust mechanism can no longer fully meet the needs of users. However, with the emergence of blockchain and smart contract technologies, they can be used to establish a decentralized mechanism to solve the above problems and lay the foundation for establishing a decentralized financial investment trust mechanism.

The blockchain is a distributed computing paradigm and decentralized infrastructure. It uses a consensus algorithm to generate and update data, uses a chain block structure to store data, uses encryption technology to verify data, and uses smart contracts to manipulate data. It has the properties of distributed, self-trust, open and transparent, tamper-proof, collective maintenance, and privacy protection. Its advantage is to solve the trust and security issues of trading [4]. The blockchain system is a free trade ecosystem based on mathematical algorithms and encryption technology. It realizes the transaction between people and machines in the form of smart contract, replacing the traditional transaction between people. The transaction is traceable and irreversible. It supports trading without a trusted third party, which is a trading paradigm superior to traditional contract [5]. The smart contract is a computer protocol that can verify and execute contracts, that is, commitments defined in digital form. Smart contracts respond to external conditions through preset instructions, complete a series of application scenario instructions, and use algorithms to ensure that contracts on the blockchain are automatically executed when conditions are triggered, so that the trading environment is standardized and orderly [6].

The characteristics of blockchain and smart contracts make the decentralized system have the following advantages [7]: (1) Fault tolerance: Since the system consists of multiple decentralized and independent nodes, it is not easily damaged as a whole. (2) Attack resistance: In a system composed of decentralized nodes, there is no so-called sensitive central node that can be subject to special attacks. The cost of attacking and the difficulty of affecting the system is also greatly increased. (3) Collusion resistance: In a decentralized multi-node system, it is much more difficult for individuals to collude to gain benefits than in a centralized system. Through blockchain and smart contract technology, the problems existing in the centralized financial investment trust mechanism can be well solved, and at the same time, it can also provide more effective supervision means for the regulatory authorities.

Aiming at the limitations of centralized system and trust mechanism, a financial investment framework based on smart contract is proposed. The framework is composed of product contract, authorization contract, and investment contract to realize a decentralized distributed financial investment trust mechanism. In this framework, the product contract provides financial products for customers. The authorization contract provides financial product authorization and contract signing authorization for investment advisors. The investment contract provides financial investment signing contracts for customers and investment advisors. The financial investment trust mechanism based on smart contract is constructed to provide a new method and solution for the establishment of trust mechanism in the online trading environment of mutual distrust, and provides a new choice for the establishment of trust mechanism of financial investment.

The roadmap of this paper is as follows. The related work is introduced in Section 2. The system structure and model are presented in Section 3. The financial investment trust mechanism based on smart contract is presented in Section 4. The proposed mechanism is implemented and verified in Section 5. The limitations and discussions are provided in Section 6. Finally, this paper is concluded.

## Related work

### Trust mechanism

In the research of trust mechanism in the financial field, there are few existing research results, which are summarized as follows. Lin et al. [8] used univariate evolutionary fuzzy clustering algorithm and perceived trust model to analyze the dynamic trust mechanism of individual consumers in rural financial market, and proved that trust, customer satisfaction, and service quality are positively correlated. Wang [9] used a computable general equilibrium model to analyze the dynamic trust mechanism of individual customers in the financial inclusion market, so as to accurately assess the trust degree of individual consumers. Brychko et al. [10] carefully researched the mediating effect of financial sector trust crisis on macroeconomic stability indicators, and concluded that the interest, credit, and monetary channels of monetary policy transmission mechanism can be used to deal with the erosion of financial sector trust crisis on macroeconomic stability conclusion. Basaran et al. [11] concluded that the effective operation and maintenance of banks are closely related to the trust and confidence generated by them and external sources by clarifying the micro-aspect of bank information production and its relationship with public trust and confidence. Cheng et al. [12] researched the trust influence mechanism of robo-advisors through a hybrid method, proposed a research model based on trust transfer theory, and clarified the relationship among trust influence factors, trust in technology, trust in suppliers, and trust in robo-advisors. Almassi [13] believed that the role of trust in financial investment is often misunderstood as financial relationships are only suitable for prudent predictive dependencies, and trust has no rational basis, which makes people not concerned about the role of trust in financial relationships, and fails to understand how Ponzi schemes fool investors.

Scholars have made many contributions to the research of trust mechanisms in the financial field. However, the trust mechanism in the financial field is currently centralized, and the drawbacks of centralized mechanism, such as single point of failure, data tampering, and information opacity, still need to be addressed. Therefore, a decentralized trust mechanism in the financial field is proposed, namely, a financial investment trust mechanism based on blockchain and smart contract, which provides a new method for researching the financial investment trust mechanism.

### Blockchain

The existing representative researches on the application of blockchain in the research field of trust mechanism are as follows. Sivaganesan [14] proposed a blockchain-based data-driven trust mechanism to detect insider attacks in (Internet of Things)-driven sensor nodes with improved message overhead. Li et al. [15] proposed a novel local trust management mechanism to solve the trust inconsistency in different regions and the false trust values generated by a group of cooperative malicious nodes, so as to effectively identify malicious behaviors. Ren et al. [16] proposed a trust establishment mechanism suitable for the distributed Internet of Things by using the blockchain technology, which reduced the number of trust domain conversions. Xiao et al. [17] proposed a blockchain-based trust mechanism to help mobile edge computing solve selfish edge attacks and forged service record attacks, which reduced response delays and saved energy. Tariq et al. [18] proposed an energy-efficient decentralized trust mechanism to prevent insider attacks in (sensor node)-driven Internet of Things, thereby improving network longevity. Zhang et al. [19] proposed a security trust mechanism based on blockchain, which realized the specific application of blockchain in quality assurance and reduced the trust cost among customers, suppliers, distributors, governments and service providers. Li et al. [20] proposed a blockchain-based service information trust mechanism to solve the trust problem between service providers and service consumers, so as to ensure that the source and storage of service information are trusted and provide reliable choices for service consumers. Lahbib et al. [21] proposed a security trust management system based on blockchain technology to provide security functions for the realization of Internet of Things scenarios, including tamper-proof and attack-resilience, reliability, and low-complexity Internet of Things applications.

Scholars have made a lot of contributions to the research of trust mechanism based on blockchain, but at present there is no research on decentralized financial investment trust mechanism, which lays the foundation for us to propose a blockchain solution of financial investment trust mechanism based on smart contract. The proposed blockchain solution solves the problem of trust building among participants who do not trust each other in the open financial investment environment, and provides a new solution for building trust system among financial investment participants.

## System structure and model

### System structure

**Fig 1:**
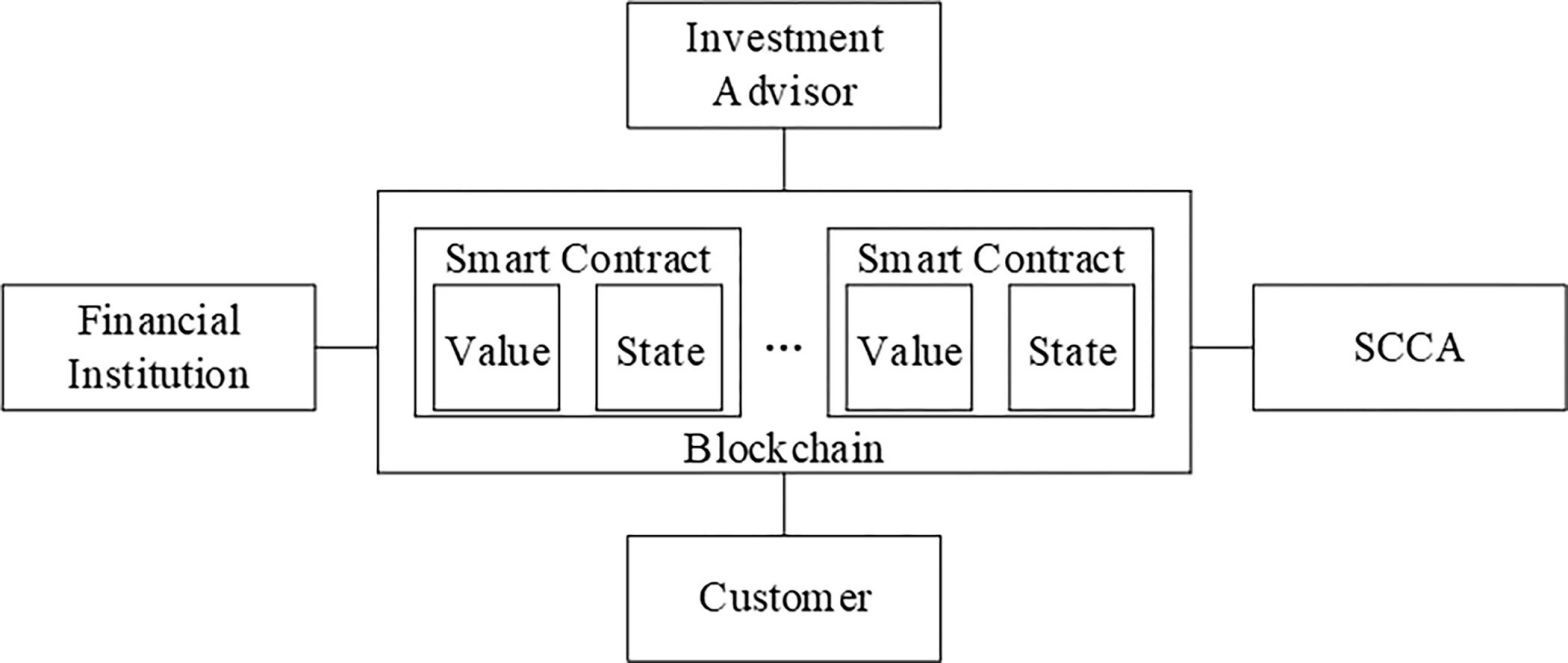
System structure.

As shown in Fig 1, the financial investment system considered in this paper is composed of blockchain, smart contracts, financial institutions, investment advisors, customers, and smart contract certification authority. Among them, smart contracts are deployed on the blockchain, and financial institutions, investment advisors, customers, and smart contract certification authority are connected through the blockchain network. The main roles on the blockchain network are explained below.

1. Financial Institution: The financial institution is the creator of smart contracts such as product contract, authorization contract, and investment contract.
2. Investment Advisor: The investment advisor is an entity that serves customers with financial investment needs.
3. Customer: The customer is an entity with financial needs and is willing to invest through the financial investment system.
4. Smart Contract Certification Authority (SCCA): The SCCA certifies the contract to ensure that the code meets the terms and conditions agreed upon among all participating entities and is in the interests of all participating entities. If the contract is attested by the SCCA, the contract address will become part of the SCCA contract and the SCCA address will be included in the contract. The SCCA can arbitrate financial investment disputes. Here, it is assumed that the SCCA will not violate the principle of fairness and justice under any circumstances.

### System model

In the decentralized financial investment system, the system has the resources (such as product contracts, authorization contracts, and investment contracts) required by participating entities. Hence, usage controls must be enforced by the system to prevent the unauthorized entities from using system resources. For example, the system must be able to prevent the request of the customer who has not paid the deposit to use the product contract, the request of the financial institution who is not the contract creator to modify the authorization contract, and the request of the investment advisor who has not obtained the authorization to sign the investment contract.

In the decentralized financial investment system model, a set of subjects *X* (they are entities that wish to obtain product authorization or investment product) and a set of objects *Y* (they are entities that own financial products). Each object *y*∈*Y* has a set of contracts *C*_*y*_ (eg, product contracts, authorization contracts, and investment contracts), and each contract *c*_*y*_∈*C*_*y*_ is associated with a set of usage rights $U_{c_{y}}$ (eg, query, update, and execute). For each subject *x* and contract *c*_*y*_, a mapping $M(x,c_{y})\subseteq U_{c_{y}}$ is defined to specify the usage rights of *c*_*y*_ granted to *x*. A usage control list mechanism is established, where each entry specifies a subject, an object contract, the operation that the subject performs on the contract, and the permissive action on the contract (eg, allow and deny). This paper aims to establish a decentralized financial investment trust mechanism based on this system model to solve the problem of trust establishment in online mutual distrust trading environment. In particular, a decentralized financial investment system framework will be proposed, which utilizes smart contracts to realize a decentralized financial investment trust mechanism.

### Financial investment trust mechanism based on smart contract

**Fig 2:**
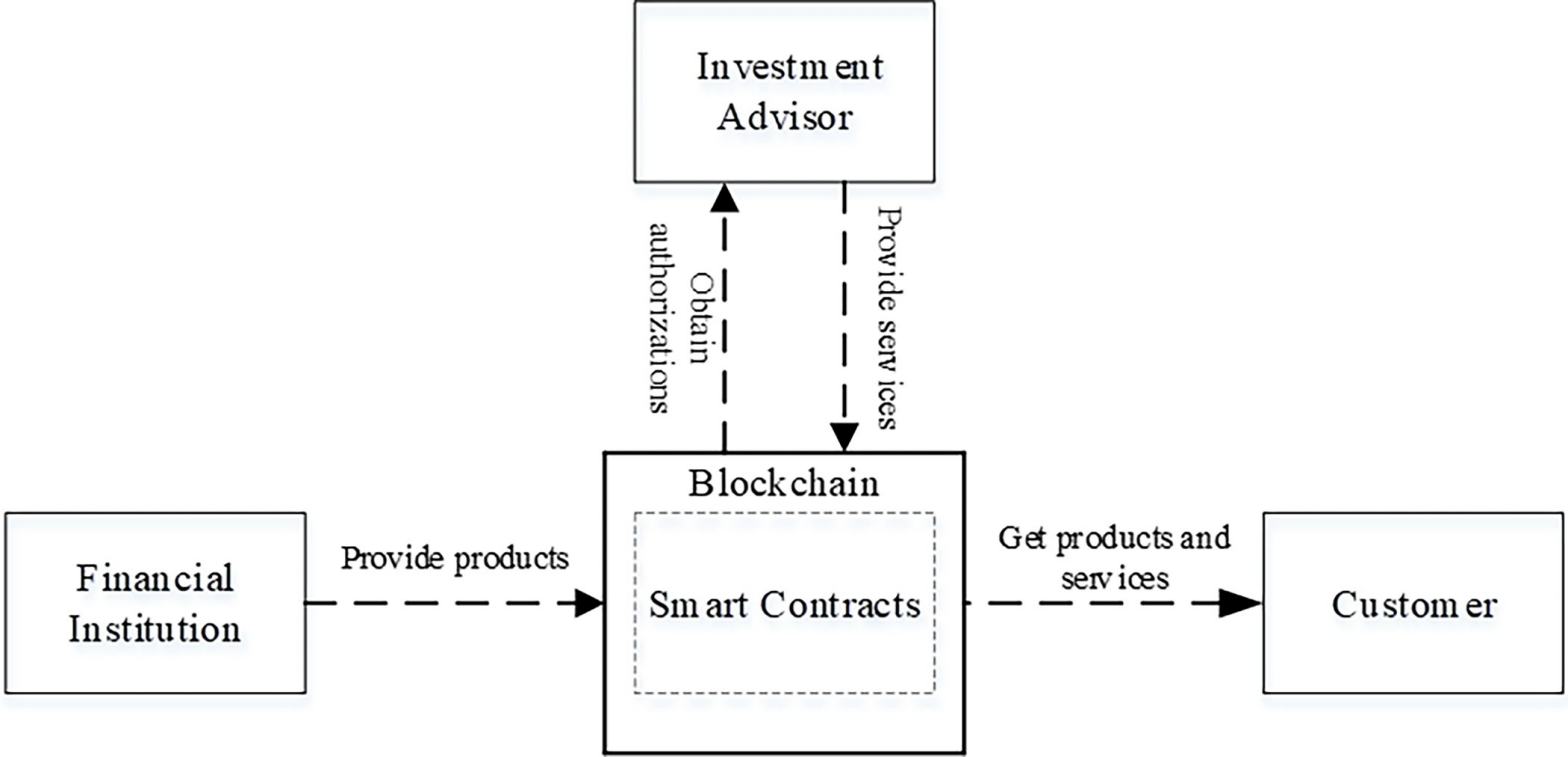
Decentralized financial investment framework.

**Fig 3:**
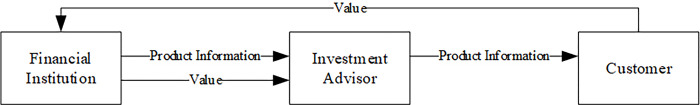
Product information flow direction and value flow direction.

The financial investment framework based on smart contract will be introduced in this section, as shown in Fig 2. It first introduces the smart contract in the framework and its role, and then explains the main functions provided by the framework. The product information flow direction of the framework: financial institution -> investment advisor -> customer, and the value flow direction of the framework: customer -> financial institution -> investment advisor, as shown in Fig 3. The financial investment trust mechanism based on smart contract is implemented based on the framework.

### Smart contracts in the framework

**Fig 4:**
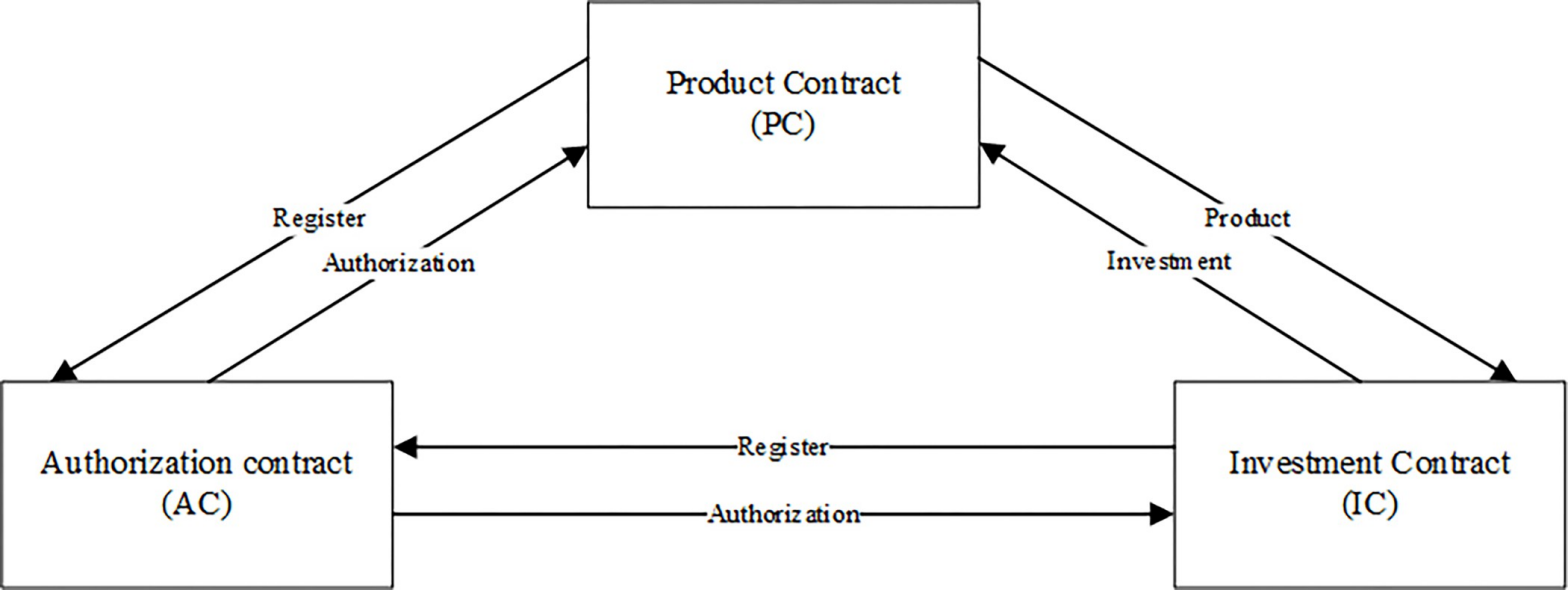
Smart contracts in the framework.

As shown in Fig 4, the proposed financial investment framework consists of product contract, authorization contract, and investment contract. The product contract is a financial product designed as a smart contract. The authorization contract is designed as a smart contract for product advisory authorization and contract signing authorization. The investment contract is a financial product investment contract designed as a smart contract. The contracts will be deployed on the Ethereum blockchain, the following is the introduction of each contract.

(1) Product contract (PC): In the financial investment framework based on smart contract, the product contract is deployed by the financial institution. The product contract provides the following functions to manage financial investment products.
  - PublishProduct(): It can only be executed by financial institutions. This function can be used to pay deposits to issue financial products.
  - AddAttribute(): It can only be executed by financial institutions to add attributes of financial products, such as name, risk level, maximum backtracking rate, etc. It takes the public key of the financial institution, the name of the financial product and the attribute of the financial product as the input parameters, and writes the output to the product contract.
  - AddPromise(): It can only be executed by financial institutions to add financial product commitments. For example, once the default clause is triggered, the deposit of the defaulting party will be automatically transferred to the account of the non-defaulting party. It takes the public key of the financial institution, the name of the financial product and the commitment of the financial product as the input parameters, and writes the output into the product contract.
  - CheckProduct(): It can only be executed by the customer to confirm whether the product is fraudulent. It takes an integer value as parameter input. For example, 1 indicates that there is no fraud in the product, and 2 indicates that there is fraud in the product.
  - SolveControversy(): It can only be executed by the SCCA to resolve product disputes. This function takes the customer’s public key and the Boolean value of the arbitration result as the parameter input, and writes the output of the result of solving the financial product fraud dispute into the product contract.
  - ChangeStatus(): It can only be executed by financial institutions to determine the operation status of product contracts.
(2) Authorization Contract (AC): In the financial investment framework based on smart contract, the authorization contract is deployed by the financial institution. The authorization contract provides the following functions to manage financial investment authorization.
  - RequestAuthorization(): It can only be executed by the investment advisor to obtain the product advisory authorization and contract signing authorization by paying the deposit.
  - AddInvestmentAdvisor(): It can only be executed by the financial institution to add investment advisors and authorize corresponding products and contracts, and then relevant information will be stored and published in the blockchain network. It takes the public key of the investment advisor, the authorization of the financial product, the authorization of the investment contract signing, and the relevant information of the investment advisor as the input parameters, and writes the output into the authorization contract.
  - RemoveInvestmentAdvisor(): It can only be executed by the financial institution to remove the investment advisor from the authorization contract and revoke the authorization of the financial product and contract signing. It takes the public key of the investment advisor as the input parameter, and writes the output of deleting the investment advisor into the authorization contract.
  - RefundInvestmentAdvisor(): It can only be executed by the investment advisor to refund the deposit paid for obtaining the financial product and contract signing authorization, and broadcast the information that the investment advisor has lost the authorization to the blockchain network.
  - RequestProduct/Signing(): It can only be executed by the customer to request financial product advisory services or sign financial investment contracts by paying a deposit.
  - ChangeStatus(): It can only be executed by the financial institution to determine the operation status of the authorization contract.
(3) Investment Contract (IC): In the financial investment framework based on smart contract, the investment contract is deployed by the financial institution. The investment contract provides the following functions to manage financial investment contracts.
  - ReleaseContract(): It can only be executed by the financial institution that can issue financial investment contracts by paying a deposit.
  - AddAgreement(): It can only be executed by the financial institution to add the content of the investment agreement to the investment contract, and store and broadcast relevant information in the blockchain network. For example, when one or more financial investment products are recommended, the constraints of the investment agreement can be the price of a product and the correlation in several product portfolios within a certain period of time. It takes the public key of the financial institution, the name of the investment agreement, and the content of the investment agreement as the input parameters, and writes the output into the investment contract.
  - CheckAgreement(): It can only be executed by the customer to check whether the agreement is fraudulent. It takes an integer value as parameter input. For example, 1 means there is no fraud in the agreement, and 2 means there is fraud in the agreement.
  - ResolveDispute(): It can only be executed by the SCCA to resolve disputes in the agreement. This function takes the customer’s public key and the Boolean value of the arbitration result as the parameter input to solve the fraud dispute in the financial investment agreement.
  - SignAgreement(): It can only be executed by the investment advisor/customer to provide an honest and trustworthy commitment by paying the deposit/investment amount to sign the financial investment contract. For example, the customer will pay the total investment amount to ensure honesty and trustworthiness to sign the financial investment contract, while the investment advisor will take 20% of the total investment amount as the honest and trustworthy deposit to sign the financial investment contract. If any default clause is triggered, the corresponding investment amount/deposit in the financial investment contract will be automatically transferred to the investment advisor/customer, which means that the default handling process does not require the intervention of a third party, thus enhancing the establishment of trust. It takes the public key of the investment advisor, the public key of the customer, the address of the financial investment contract, and the deposit/investment amount of the investment advisor/customer as the input parameters, and writes the output into the investment contract.
  - ChangeStatus(): It can only be executed by the financial institution to determine the operation status of the investment contract.

### Financial investment process

When the customer has financial investment needs, he can request financial investment services from the investment adviser. The financial investment service process is as follows.

- Customer advisory. Customer *c* wants to buy financial product *p*.
- Investment advisor declaration. The investment advisor *i* claims to have the corresponding product authorized by the financial institution *f*.
- Customer check information. In view of the declaration of *i*, *c* asks the *i*.EOA of the product authorized by *f*. To confirm the information related to *i*.EOA, *c* will check the public media that releases the authorization contract. *c* can check the relevant information of *i*.EOA by accessing the authorization contract to confirm that *i*.EOA does have the product authorization of *f*. Then *c* will verify whether it is the real owner of *i*.EOA by challenging *i*.
- Customer challenge. *c* arbitrarily selects the information data *md* to request *i* to sign it.
- Investment advisor response. *i* uses the public key *i*. EOA and the private key *i*.EPK to sign *md*. Signature is defined by *Signing* = *Sign* (*i*.EOA, *i*.EPK, *md*). This means that *md* can only be signed when *i* is the real owner of *i*.EOA and *i*.EPK. After signing, *i* sends the *Signing* back to *c*.
- Customer validation response. After *c* receives the signature of *i* to *md*, *c* can use the function *ResponseVerify* (*i*.EOA, *md*, *Signing*) [22] to verify.
- Investment advisor supply product. If the identity and authorization of the investment advisor are verified, the investment advisor can provide *c* with the access address and interactive interface of *p*.
- Customer checking product. *c* can access and check *p* through the received address and interface. If *p* has a fraud problem, *c* can submit the dispute to SCCA for arbitration. If *p* has no fraud problem, *c* can continue to sign the contract in the next step.
- Customer checking investment contract. When *c* checks that *p* has no fraud problem and wants to sign an investment contract with *i*, *c* can request *i* to sign a financial investment contract. *i* can send the access address and interactive interface of the investment contract to *c* for *c* to access and check the investment contract. If there is fraud in the investment contract, the dispute can be submitted to SCCA for arbitration. If there is no fraud in the investment contract, *c* and *i* can continue to sign the investment contract in the next step.
- Both parties signing investment contract. After confirming that there is no problem with the financial product and the agreement, *c* and *i* can sign the investment contract. After the investment contract is signed, the financial investment process is completed.

**Fig 5:**
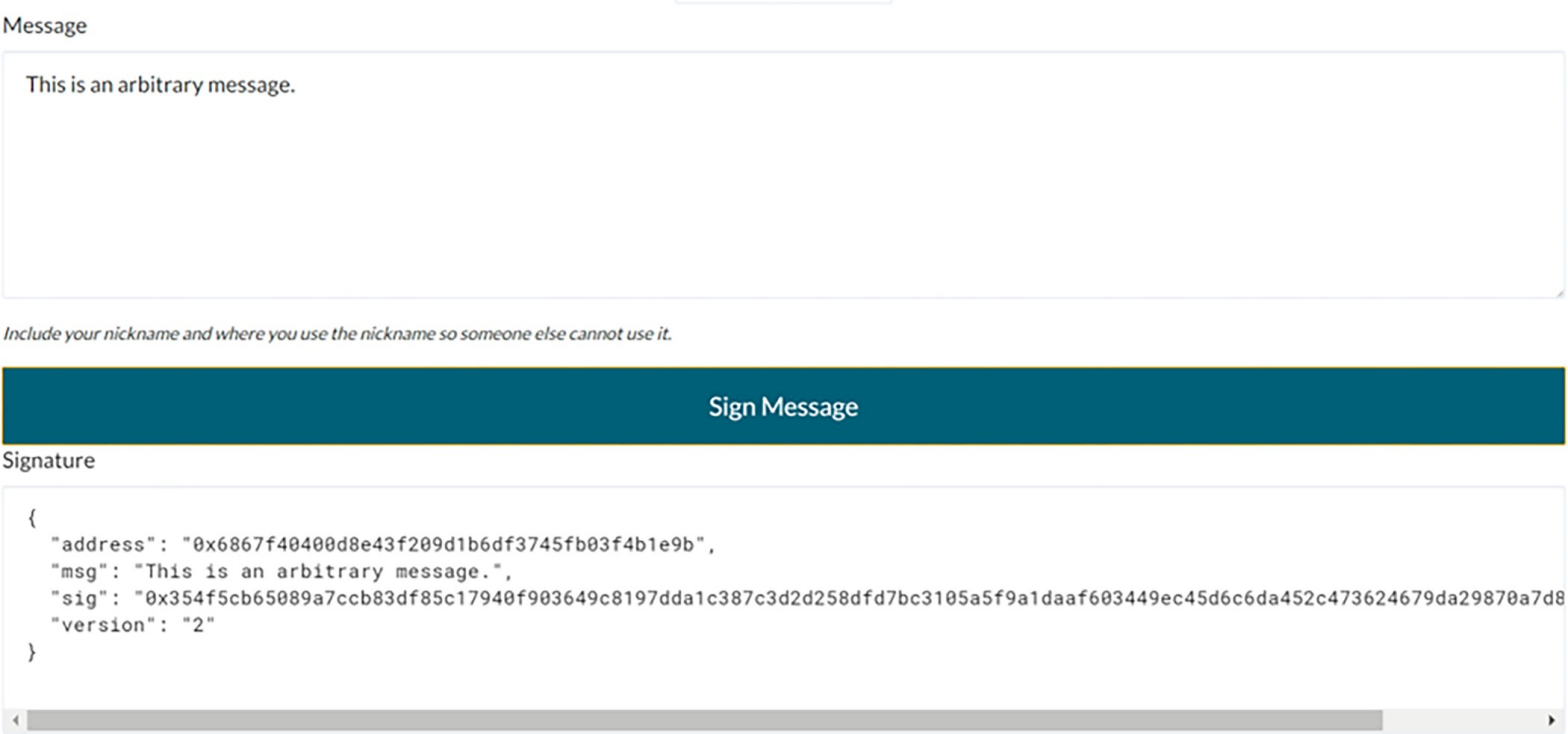
Signature.

**Fig 6:**
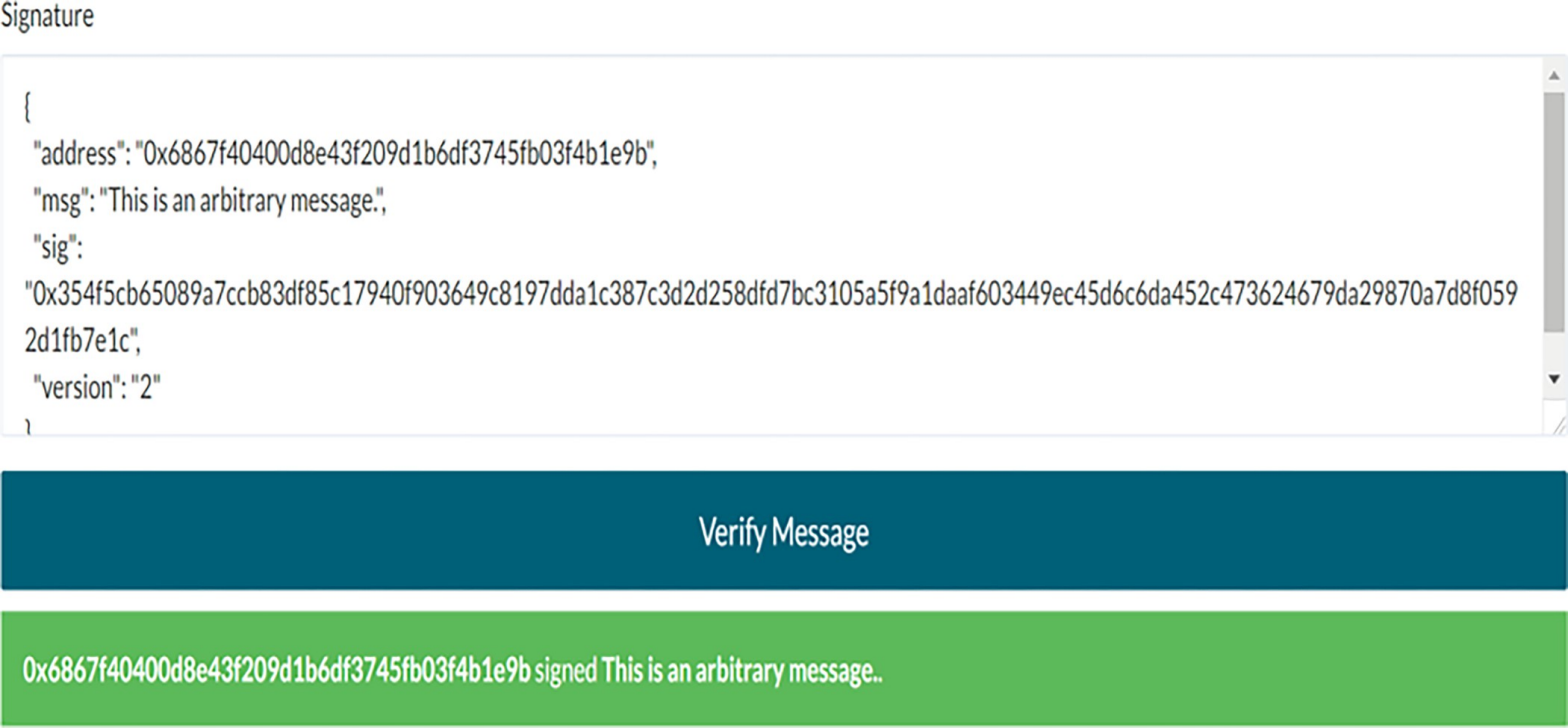
Signature verification.

It should be noted here that EOA represents the public key (that is, the address) of the entity, and EPK represents the private key of the entity. Furthermore, *eth*.*sign*(address, web3.sha3(message)) function [23] can be used for signature creation. Its advantage is that users can sign message data without disclosing the private key. Figs 5 and 6 show an example of signature and signature verification respectively, which can prove the ownership of the user’s public key.

## Implementation and verification of the proposed mechanism

To demonstrate the application of the proposed financial investment framework and verify the correctness and effectiveness of the financial investment trust mechanism based on smart contract, the implementation and verification of the proposed mechanism is provided in this section. The hardware and software used in the research will be introduced first, how to implement the proposed mechanism through the usage control based on the proposed framework will be presented later, and some experimental results will be presented at the end.

### Hardware and software

The case of five laptops with moderate performance is considered. The main hardware specifications of the laptop are shown in Table 1. Two laptops are used as the subject (i.e. customer and investment advisor), and one laptop is used as the object (i.e. financial institution). The remaining two laptops are used as validating and bookkeeping nodes (i.e. as miners for mining).

**Table 1:** The main hardware specifications of laptop.

Hardware name	Specification parameter
**CPU**	Intel(R) Core(TM) i5-5200U CPU @ 2.20GHz 2.19GHz
**RAM**	8.00 GB
**System Type**	Win 10 Pro (64-bit)
**Hard Disk**	1TB
**Graphics Card**	Intel(R) HD Graphics 5500

**Fig 7:**
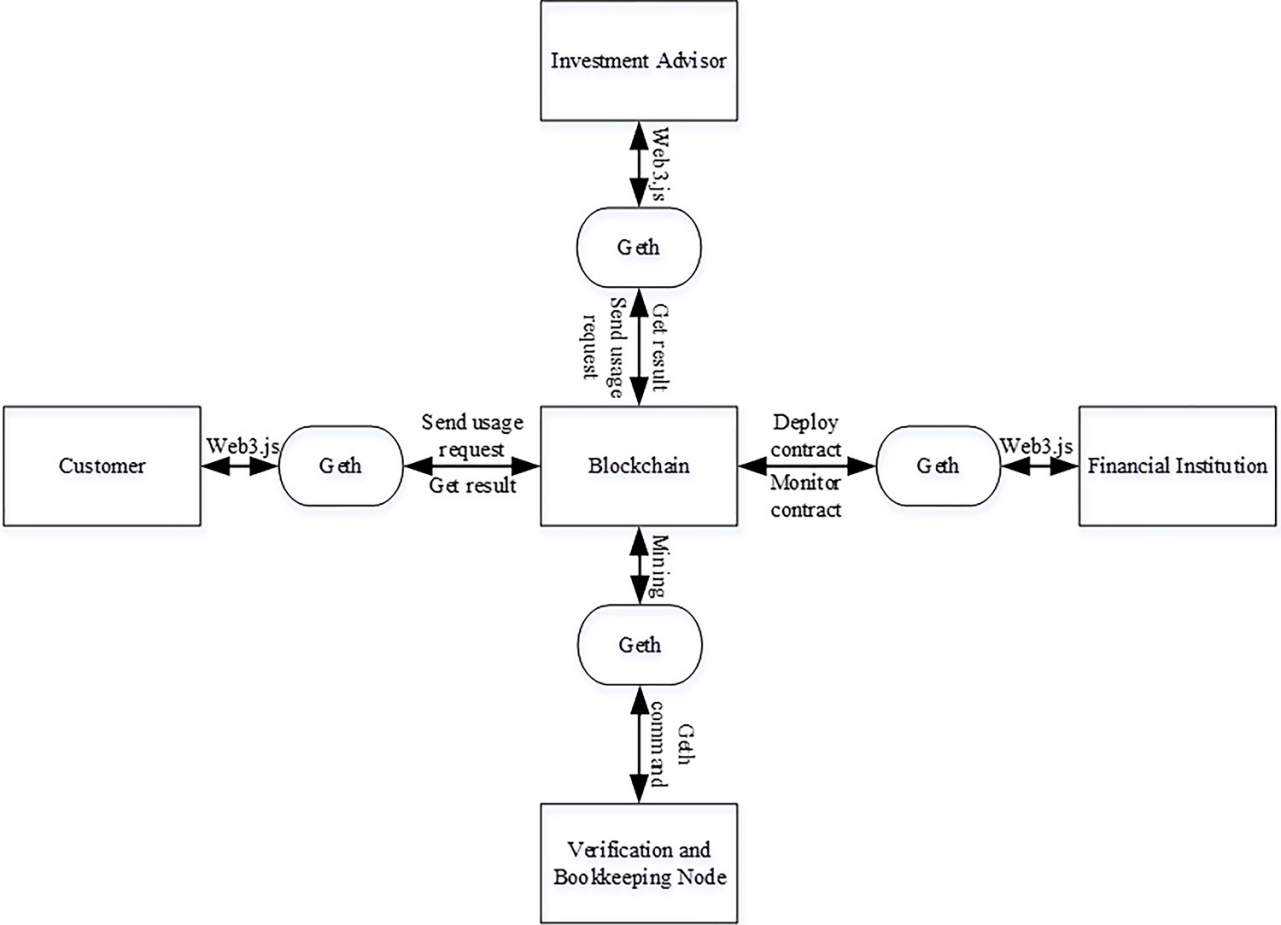
The composition and interaction of blockchain nodes.

A Geth client [24] (i.e. an Ethereum node) is installed on each laptop, so that the laptop can join the Ethereum blockchain as a node. Each laptop can create an Ethereum account through the Geth client. These laptops with Ethereum accounts can be converted into blockchain nodes and configured as a private blockchain network, as shown in Fig 7. It is worth noting that the verification and bookkeeping nodes are full nodes of Ethereum, and the subject and object nodes are light nodes to facilitate usage control.

For smart contract writing and compilation, the Remix IDE [25] will be used. It is a browser-based IDE for writing, compiling and deploying smart contracts. The programming language for writing smart contracts will be Solidity [26]. It is the most popular smart contract programming language in the world. After the smart contract is written and debugged, it will be deployed to Ethereum Wallet [27]. Furthermore, on the object side, the official Ethereum JavaScript API (i.e. Web3.js [28]) will be adopted. The object side uses Web3.js to interact with the corresponding Geth client through an HTTP connection for deploying the compiled smart contract and monitoring the status of smart contract (e.g. the result of usage control). On the subject side, the subject side also adopts Web3.js to interact with the corresponding Geth client, so as to send a usage request to the smart contract by sending a transaction, and receive the usage control result from the smart contract. On the verification and bookkeeping node side, the node mines on the blockchain through the Geth command to verify the transaction and bookkeep the legal transaction into the blockchain.

### Implementation

**Fig 8:**
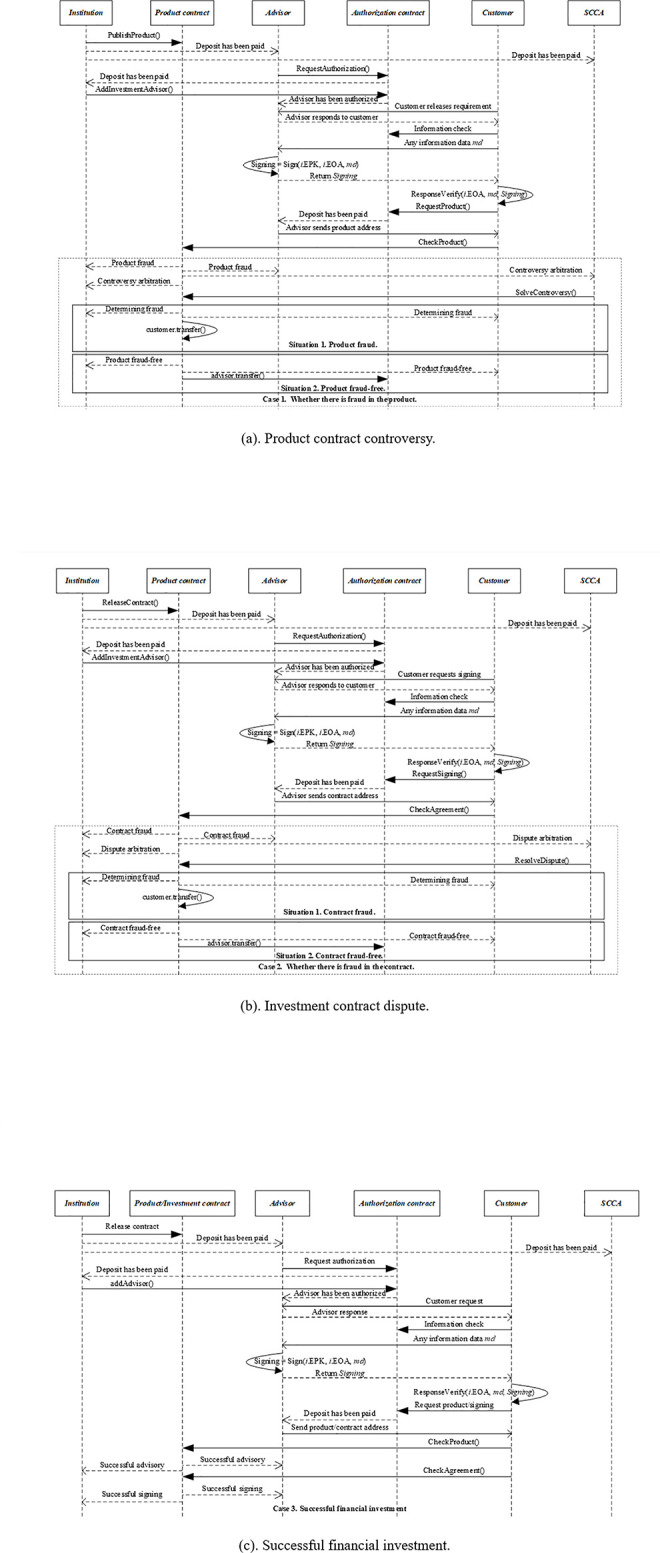
Financial investment sequence diagram. (a). Product contract controversy. (b). Investment contract dispute. (c). Successful fmancial investment.

The customer broadcasts the demand for the financial product through the blockchain network. The investment advisor can respond to the customer through the Ethereum address. The customer verifies the authenticity of the response by accessing the authorization contract, and confirms whether the investment advisor is the real owner of the Ethereum address by verifying the digital signature of the investment advisor. After the identity and authorization of the investment advisor are verified, if the customer agrees with the terms and conditions of the product contract, he/she can request to access and check the product contract. If there is fraud in the product contract, the investment advisor can apply for controversy arbitration. If there is no fraud in the product contract, then the customer can request to access and verify the investment contract. If there is fraud in the investment contract, the customer can apply for dispute arbitration. If there is no fraud in the investment contract, the customer can request to sign the investment contract. After the investment contract is signed successfully, it means that the financial investment is successful. The sequence diagram of financial investment is shown in Fig 8.

The sequence diagram describes the complete process from the financial needs of customer to the completion of financial investment, as well as the functions and events invoked in the smart contract. Wherein, Case 1 describes the process by which the product contract may be fraudulent and the controversy arbitration is enforced. Case 2 describes the process in which the investment contract may have fraud and the dispute arbitration is executed. Case 3 describes the process that the customer is satisfied with both the product contract and the investment contract, there is no dispute between the trading parties, and the financial investment is successful.

Next, the important algorithms used in the smart contract code will be described in detail:

(1) Request financial investment service algorithm

The customer needs to request the financial investment service by paying a deposit to the authorization contract. This is to ensure that the customer is honest and trustworthy. The investment advisor will first provide the financial product advisory service, and will send the address and interface of the product contract to the customer, so that the customer can access and check whether the product contract is appropriate. If the customer believes that there is no problem with the product contract and is willing to sign the investment contract, the investment advisor will provide the contract signing service and send the address and interface of the investment contract to the customer, so that the customer can access and verify whether the contract is appropriate. Algorithm 1 shows the algorithm that the customer requests the financial investment service.

**Algorithm 1. Request financial investment service.**

**Input:** EOA, depositValue, authorizationStatus, customerStatus

**Output:** product.EOA/contract.EOA

**1:** EOA ← Ethereum address.

**2:** depositValue ← deposit value.

**3:** authorizationStatus ← authorization contract status.

**4:** customerStatus ← customer status.

**5: if** msg.value = depositValue **then**

**6:   if** authorizationStatus = waiting for customer **then**

**7:** customer.EOA.send(msg.value). //Customer has paid the deposit.

**8:** customerStatus ← customerPaid. //customerStatus status is customerPaid.

**9:** The notice that the customer has paid the deposit is created.

**10:** The notice that the address and interface of product/investment contract have been sent to the customer is created.

**11:   else**

**12:** Restore the authorization contract status and display an error.

**13:   end if**

**14: end if**

(2) Successful financial investment algorithm

After the customer accesses and verifies the product/investment contract through the address and interface of the product/investment contract, if the customer confirms that the product/investment contract is qualified, it can complete the signing of the financial product investment contract through the investment contract, and the financial investment is successful. Algorithm 2 shows the algorithm that the financial investment is successful.

**Algorithm 2. Successful financial investment.**

**Input:** EOA, customerStatus, customerPaid, SCCAReceivedArbitration

**Output:** Successful financial investment

**1:** EOA ← Ethereum address.

**2:** customerStatus ← customer status.

**3:** customerPaid ← customerStatus value.

**4:** SCCAReceivedArbitration ← customerStatus value.

**5: if** customerStatus = = customerPaid or customerStatus = = SCCAReceivedArbitration **then**

**6: if** customerStatus = = customerPaid **then**

**7:**  The notice that the financial investment has been successful is created.

**8:   else**

**9: if** customerStatus = = SCCAReceivedArbitration **then**

**10:**    Execute SolveControversy()/ResolveDispute().

**11:**    Turn to Algorithm 3/4.

**12: else**

**13:**    Restore the product/investment contract status and display an error.

**14: end if**

**15:   end if**

**16: else**

**17:**   Restore the product/investment contract status and display an error.

**18: end if**

(3) Product contract fraud arbitration algorithm

If the customer believes that the product contract is fraudulent after visiting and checking the product contract, he/she can apply to the SCCA for arbitration. The SCCA will determine whether there is fraud in the product contract by visiting and verifying the product contract and referring to the corresponding case. If the arbitration result is that there is no fraud in the product contract, the customer will be punished accordingly. If the arbitration result is that there is fraud in the product contract, the financial institution and the investment advisor will be fined. Algorithm 3 shows the algorithm that the product contract fraud is arbitrated.

**Algorithm 3. Product fraud arbitration**.

**Input:** EOA, result, customerStatus, controversyArbitration

**Output:** Arbitration result.

**1:** EOA ← Ethereum address.

**2:** result ← Arbitration result.

**3:** customerStatus ← customer status.

**4:** controversyArbitration ← customerStatus value.

**5: if** customerStatus = = controversyArbitration **then**

**6: if** result = = false **then**

**7:**  result ← There is no fraud in the product.

**8:**  Execute PaymentAndSettlement().

**9:   else**

**10:** result ← There is fraud in the product.

**11:** Execute PenaltyandSettlement().

**12:   end if**

**13: else**

**14:**   Restore the product contract status and display an error.

**15: end if**

(4) Investment contract fraud arbitration algorithm

If the customer believes that the investment contract is fraudulent after visiting and checking the investment contract, he/she can apply to the SCCA for arbitration. The SCCA will determine whether there is fraud in the investment contract by visiting and verifying the investment contract and referring to the corresponding case. If the arbitration result is that there is no fraud in the investment contract, the customer will be punished accordingly. If the arbitration result is that there is fraud in the investment contract, the financial institution and the investment adviser will be fined. Algorithm 4 shows the algorithm that the investment contract fraud is arbitrated.

**Algorithm 4. Contract fraud arbitration.**

**Input:** EOA, result, customerStatus, disputeArbitration

**Output:** Arbitration result.

**1:** EOA ← Ethereum address.

**2:** result ← Arbitration result.

**3:** customerStatus ← customer status.

**4:** disputeArbitration ← customerStatus value.

**5: if** customerStatus = = disputeArbitration **then**

**6: if** result = = false **then**

**7:**  result ← There is no fraud in the contract.

**8:**  Execute PaymentAndSettlement().

**9:   else**

**10:** result ← There is fraud in the contract.

**11:** Execute PenaltyandSettlement().

**12:   end if**

**13: else**

**14:**   Restore the investment contract status and display an error.

**15: end if**

### Experiment

The source code of the product contract, authorization contract, and investment contract can be obtained on [29]. Based on hardware, software, and code, the experiment is carried out, and the financial investment framework based on smart contract is proved to be feasible.

(1) Blockchain network construction

**Fig 9:**
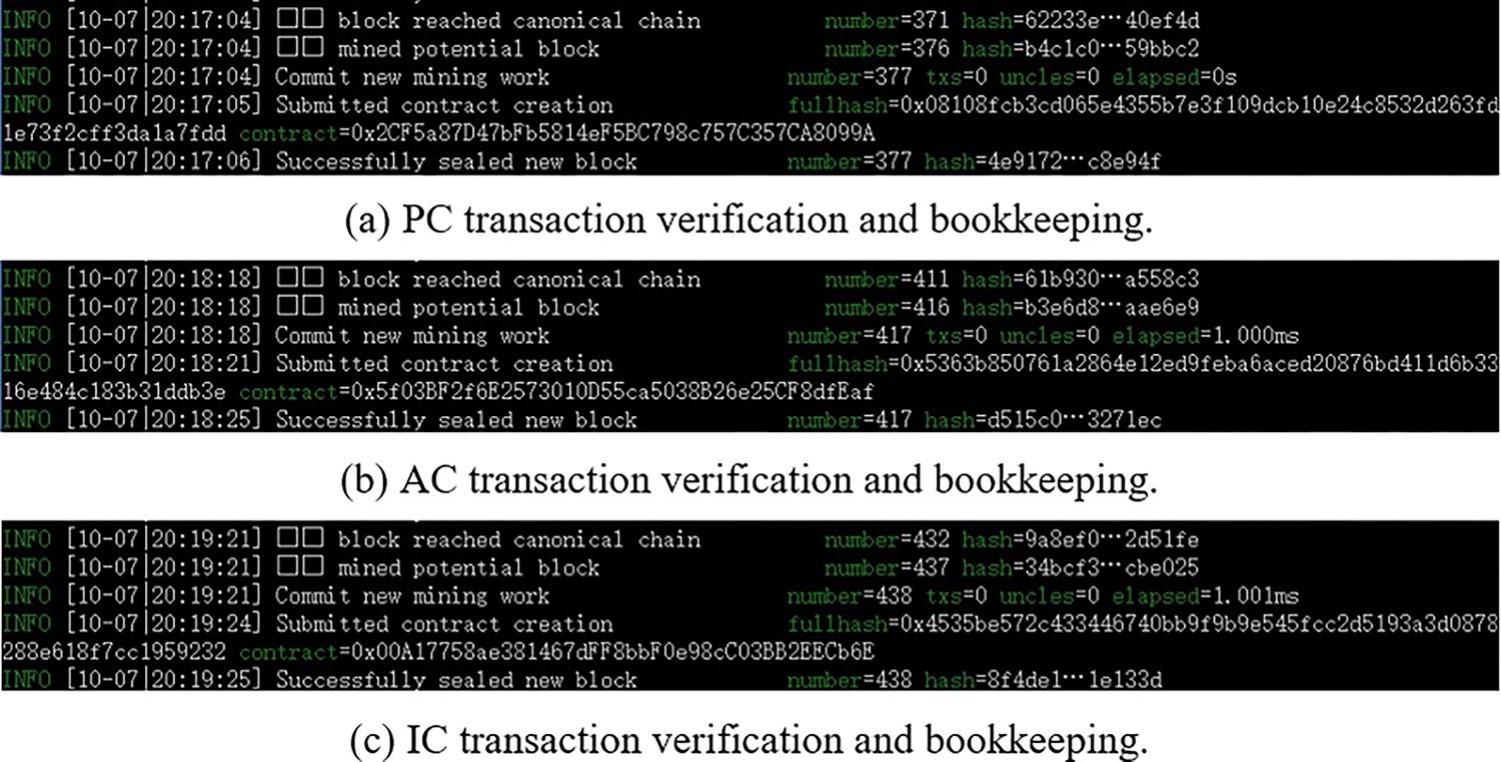
Node verification and bookkeeping. (a) PC transaction venfication and bookkeeping. (b) AC transaction venfication and bookkeeping. (c) IC transaction verification and bookkeeping.

Geth is used to build the blockchain network, and Web3.js is used to interact with Geth. A genesis block file will be created. There is only one genesis block in the same blockchain network, and only nodes originating from the same genesis block can communicate normally. A single node can create different accounts, and then use different accounts for transactions. Multiple nodes can be connected through the admin.addPeer ("node’s enode") command. The node verification and bookkeeping in the blockchain network is shown in Fig 9.

(2) Smart contract deployment

**Fig 10:**
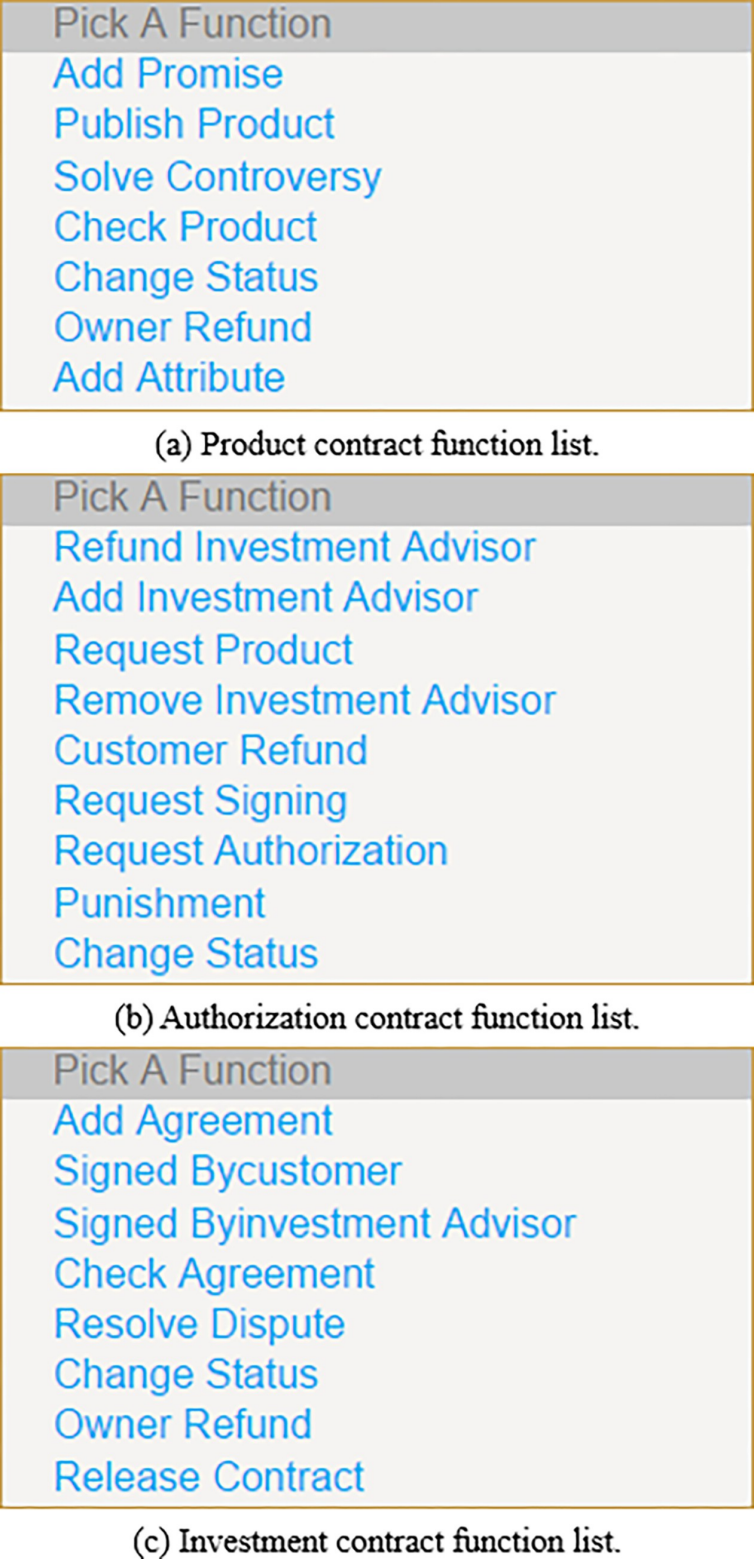
Smart contract function list. (a) Product contract function list. (b) Authorization contract function list. (c) Investment contract function list.

After Remix IDE compiles and debugs the smart contract written in Solidity language, the smart contract will be deployed in Ethereum Wallet. The function list of the product contract, authorization contract, and investment contract is shown in Fig 10. In addition, the addresses of smart contracts are as follows:

The PC’s Ethereum address:

                                             “0x2CF5a87D47bFb5814eF5BC798c757C357CA8099A”
The AC’s Ethereum address:
                                             “0x5f03BF2f6E2573010D55ca5038B26e25CF8dfEaf”
The IC’s Ethereum address:
                                             “0x00A17758ae381467dFF8bbF0e98cC03BB2EECb6E”

(3) Main function verification

By simulating the main functions of smart contracts, the simulation of product contract, authorization contract and investment contract is realized. By generating event occurrence diagram and combining with the process from request to completion of financial investment, the effectiveness of the model and algorithm is verified. In the Ethereum blockchain, the Ethereum address of the participating entity is as follows:

The financial institution’s Ethereum address:

                                             “0x8020A2bd757d33e6Fd5F5212bF6F7b8025563Cb4”
The investment advisor’s Ethereum address:
                                             “0x75afF96CC8E304a943237Cd933E0fdC4B212d745”
The customer’s Ethereum address:
                                             “0x5BFc90dF1DE9355F818ED99733B597268c90C01E”
The SCCA’s Ethereum address:
                                             “0xd8BE8bF315475A19737cd710dE4deAE466706991”
  (1) Request financial investment

**Fig 11:**
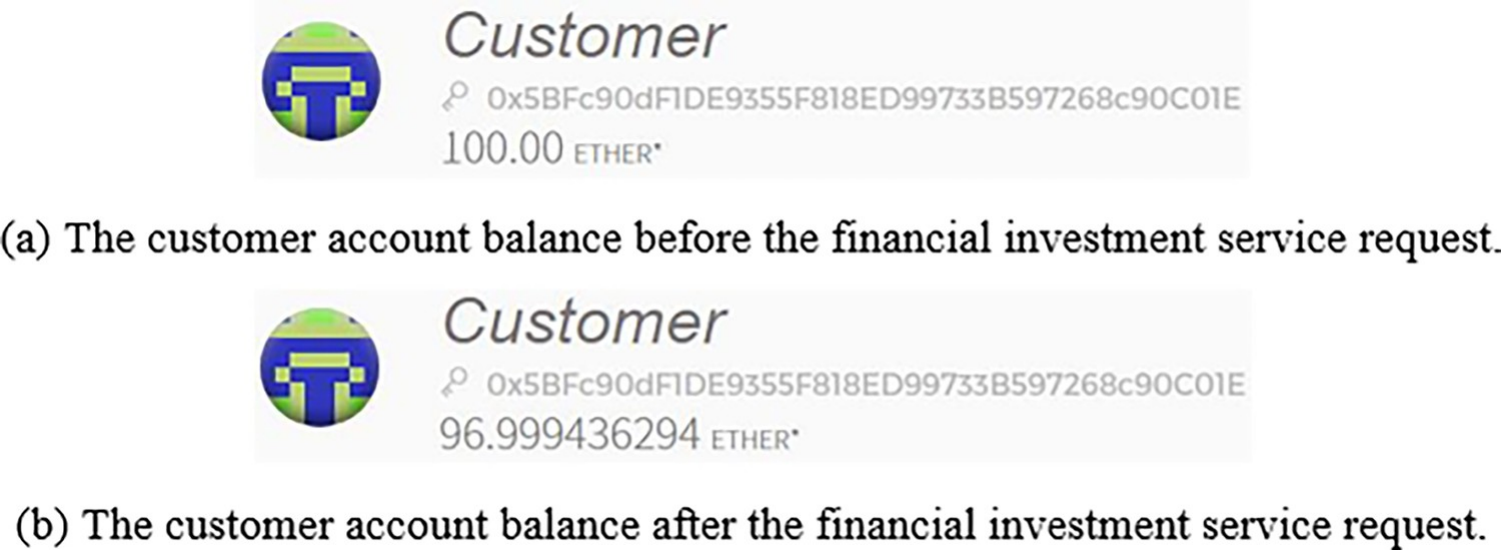
Customer financial investment service request. (a) The customer account balance before the fínancial investment Service request. (b) The customer account balance after the fínancial investment Service request.

The customer uses the RequestProduct() function to send a request for financial investment service to the authorization contract. Fig 11(A) shows the customer account balance before the financial investment service request. Fig 11(B) shows the customer account balance after the financial investment service request.

From the results shown in Fig 11, it can be seen that the "authorization contract" and "request financial investment service algorithm" are feasible and effective.

(2) Dispute resolution

**Fig 12:**
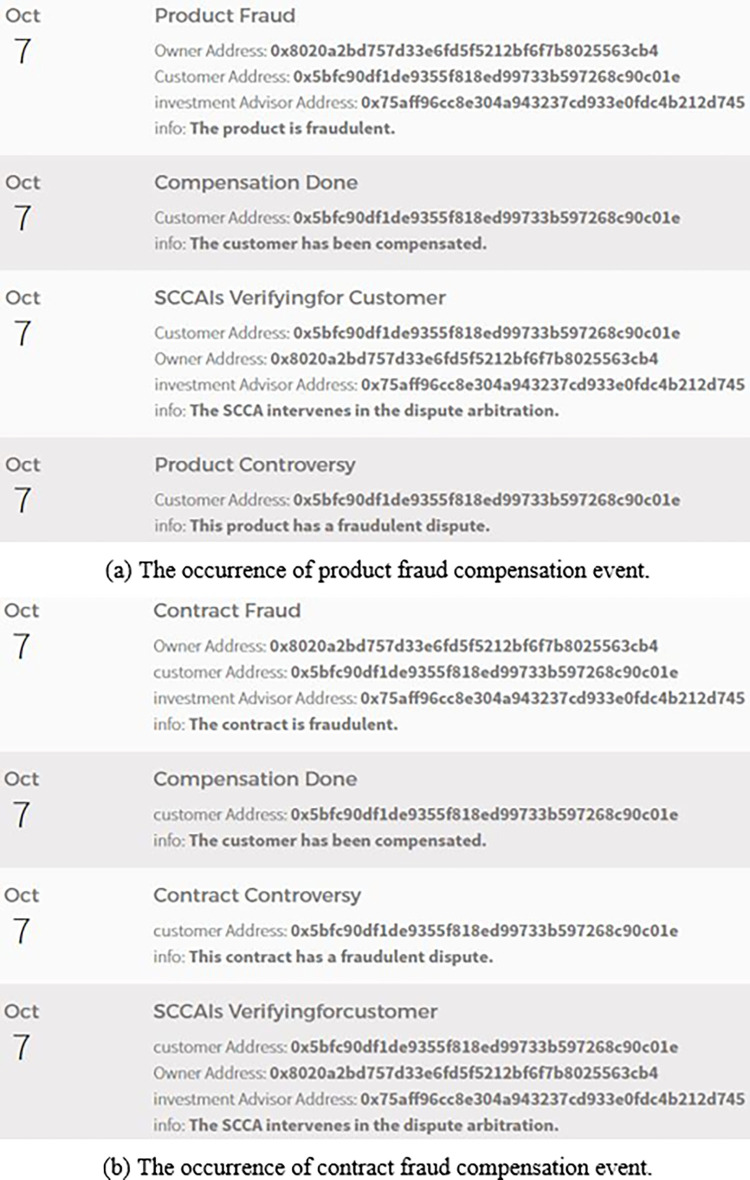
Dispute resolution. (a) The occurrence of product fraud compensation event. Contract Fraud. (b) The occurrence of contract fraud compensation event.

If the customer uses the CheckProduct()/CheckAgreement() function to confirm that the product/contract has fraud, the SCCA will intervene in dispute arbitration. If the arbitration result is "fraud exists in the product/contract", the product/investment contract will automatically complete the settlement of compensation to the customer. If the arbitration result is "no fraud exists in the product/contract", the customer will also be fined for malicious reporting. Fig 12(A) shows the occurrence of product fraud compensation event. Fig 12(B) shows the occurrence of contract fraud compensation event.

It can be seen from the results shown in Fig 12 that "product contract and investment contract", "product contract fraud arbitration algorithm and investment contract fraud arbitration algorithm" are feasible and effective.

(3) Successful financial investment

**Fig 13:**
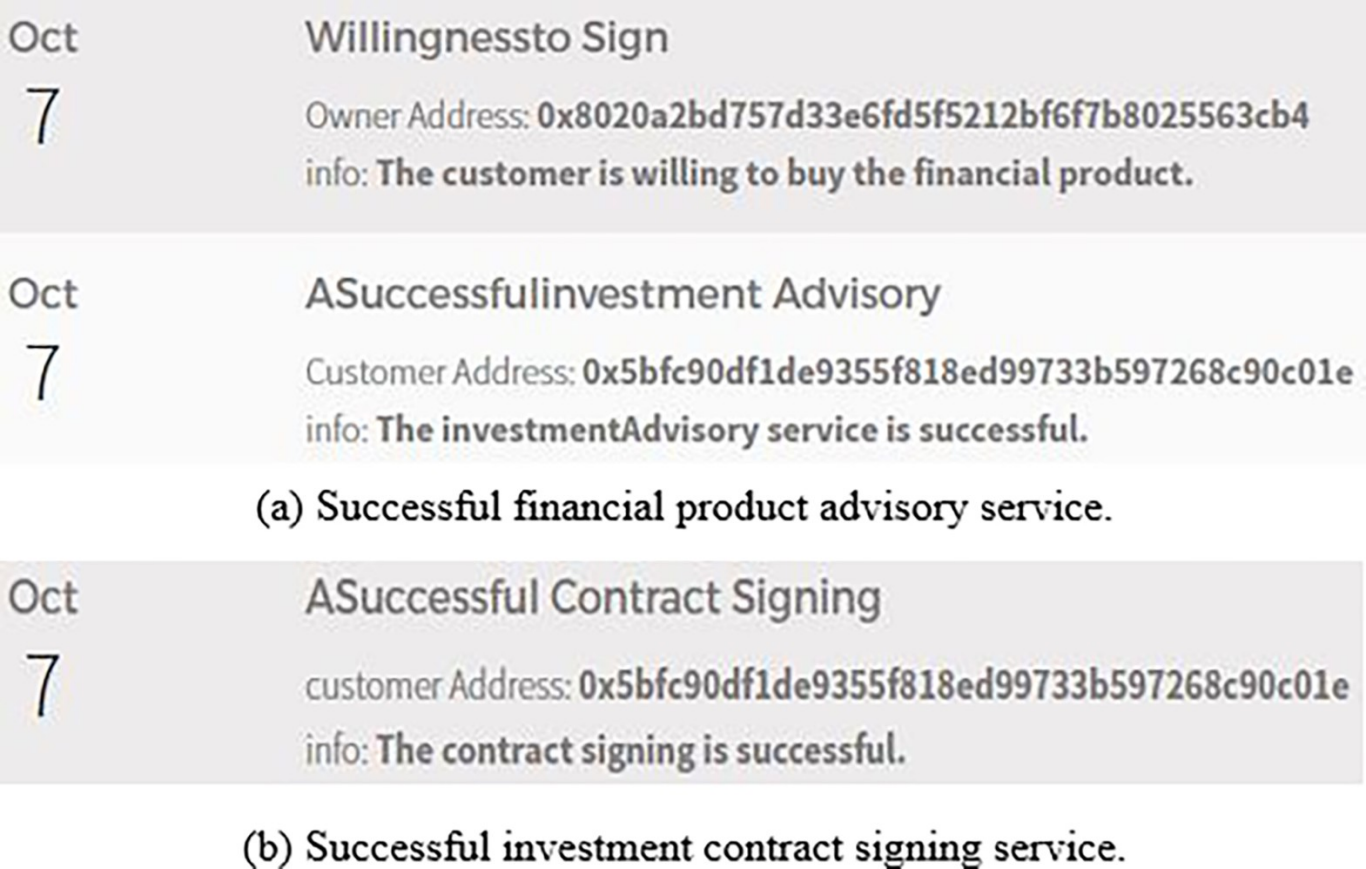
Successful financial investment. (a) Successful financial product advisory sen-ice. (b) Successful investment contract signing sen’ice.

The customer uses the CheckProduct()/CheckAgreement() function to confirm whether the financial product advisory service and the investment contract signing service are successful. Fig 13(A) shows the success of financial product advisory service. Fig 13(b) shows the success of the investment contract signing service.

It can be seen from the results shown in Fig 13 that the "Successful financial investment algorithm" is feasible and effective.

## Limitations and discussions

### Reasons based on smart contract

Information asymmetry exists in the centralized financial investment mechanism, which easily leads to distrust. A smart contract is a computer program that runs automatically and faithfully. It has the function of processing and storing information data, as well as the function of asset control and processing. While being deployed on the blockchain, smart contracts have the characteristics of decentralization, data tamper-proof, information openness and transparency, and transaction traceability, which can solve the problem of financial investment distrust caused by information asymmetry. This is why smart contracts (product contracts, authorization contracts, and investment contracts) have been selected to build a trust mechanism for financial investment. This provides a new choice for the establishment of financial investment trust mechanism.

Aiming at the problem of distrust caused by information asymmetry, a solution of financial investment trust mechanism based on smart contract is proposed. This solution is divided into three phases. In the first stage, "authorization contract" and "Request financial investment service algorithm" were designed to achieve the goal of "financial investment request". In the second stage, "product contract", "investment contract" and "investment contract fraud arbitration algorithm" were designed to achieve the goal of "successful financial investment". In the third stage, "Product contract fraud arbitration algorithm" and "Investment contract fraud arbitration algorithm" are designed to achieve the goal of "product and contract dispute resolution". Three stages were completed and the problem of distrust in financial investment was solved. This provides a new method and choice to solve the problem of distrust caused by information asymmetry and establish a financial investment trust mechanism.

### Safety analysis

- Authentication and authorization: The functions of the product, authorization, and investment contract can only be performed by the specific authorized participating entity. This ensures the authenticity of the identity, information content, and transaction time of the financial investment participating entity. This effectively ensures the authenticity and credibility of the financial investment trust mechanism.
- Integrity: All information and data exchanged between blockchain nodes are tamper-proof, which is an important feature of blockchain. This makes the financial investment data of customers, financial investment service data of investment advisors, and financial investment contract data of financial institutions tamper-proof. This ensures that the entire financial investment information is complete and has not been tampered with or forged. This effectively ensures the data integrity of the financial investment trust mechanism.
- Confidentiality: No entity can explain the communication between any other parties on the blockchain, and unauthorized entities cannot access data that requires authorization. This confidentiality is an important feature of the blockchain and can be achieved through encryption. This ensures the confidentiality of the data and information exchanged in the whole financial investment, so that they are not known by entities unrelated to financial investment other than participating entities. This effectively realizes the communication and data confidentiality of the financial investment trust mechanism.
- Non-repudiation: Communication and events are important components of the distributed ledger log on the blockchain. It records the originator of any operation on the blockchain, and no entity can tamper with its records. Therefore, no entity can deny any operation initiated by it. This ensures that financial investment participating entities cannot deny the fact that they have participated in transactions, and it provides a piece of credible evidence for possible future transaction disputes. This effectively realizes the transparency and traceability of the financial investment trust mechanism.

### Advantages and disadvantages

A trust mechanism for financial investment based on smart contract is proposed. In this mechanism, financial products are designed as product contracts, financial service qualifications are designed as authorization contracts, and financial contracts are designed as investment contracts. The centralized trust mechanism cannot eliminate the influence of factors that easily cause information asymmetry, such as data tamper-proof and transparency, which easily leads to distrust. In contrast, the characteristics of blockchain, such as decentralization, data tamper-proof, and information transparency, help to build a financial investment trust mechanism based on smart contracts on the basis of eliminating information asymmetry, so that it is easy to establish trust between traders. In addition, the proposed product/contract dispute resolution mechanism and algorithm can obtain accurate arbitration results, and simply and effectively resolve fraud disputes existing in the product/contract.

However, since this paper proposes a new trust mechanism for financial investment based on smart contracts, the maturity of this mechanism needs to be further verified in practice. Furthermore, although product, authorization, and investment contracts are all designed as smart contracts, they can only be designed as smart contracts if the rules are clear and are not affected by subjective factors. A possible solution to this limitation is to further strengthen the intelligence of smart contracts. Moreover, the value of cryptocurrency is volatile and uncertain, which will lead to the value of wealth management investment that is also volatile and uncertain. One possible way to address this limitation is to introduce a cryptocurrency that is linked to the value of the subject asset, such as fiat money, to ensure that the value of the cryptocurrency is consistent. Finally, in product/contract fraud disputes, the SCCA may make incorrect rulings contrary to the principles of fairness and justice. One possible way to address this limitation is to establish a scientifically sound system of rewards and punishments to ensure that the SCCA makes correct decisions.

## Conclusion

A financial investment trust mechanism based on smart contract is proposed to solve the problem of financial investment distrust. First, the trust mechanism of financial investment is constructed to realize the financial investment solution from "requesting financial investment", "signing investment contract" to "resolving disputes". Next, the process of financial investment is controlled by designing algorithms of "requesting financial investment service", "successful financial investment", "product contract fraud arbitration" and "investment contract fraud arbitration". Furthermore, smart contracts are written and debugged by Solidity and Remix IDE. Moreover, smart contracts are verified by Geth, Web3.js, and Ethereum Wallet. Finally, the security features of the solution such as integrity, confidentiality, and non-repudiation are discussed.
